# Supplementary material for: Assessing the impact of variance heterogeneity and misspecification in mixed-effects location-scale models
Source: BMC Med Res Methodol. 2026 Jan 20;26:53. doi: 10.1186/s12874-025-02755-3 (PMC12961882; doi:10.1186/s12874-025-02755-3)
Supplement: Supplementary file 1 — Supplementary Material 1. [file 12874_2025_2755_MOESM1_ESM.pdf]

Supplementary Material

Assessing the impact of variance heterogeneity and misspecification in mixed-effects location-scale models

Vincent Jeanselme[1,2], Marco Palma[1,3*], Jessica Barrett[1]

[1]MRC Biostatistics Unit, University of Cambridge, UK.
[2]Department of Biomedical Informatics, Columbia University, USA.
[3]Population, Policy and Practice Research and Teaching Department, UCL Great Ormond Street Institute of Child Health, UK.

*Corresponding author(s). E-mail(s): marco.palma@ucl.ac.uk;

Appendix A Why do we expect a biased location model to have a larger scale error?

Let us simplify the problem by considering the following generative process:

$$Y = \beta X + \epsilon$$

with $\epsilon \sim \mathcal{N}(0, \omega)$.

A biased scale model can be expressed as:

$$Y = (\beta + \delta)X + \epsilon'$$

with $\epsilon' \sim \mathcal{N}(0, \omega')$ and δ the bias from the true coefficient.

Computing the difference between the generative process and the biased model results in:

$$\epsilon' = \epsilon - \delta X$$

with $X \sim MVN(0, \Sigma)$. And therefore, one can express the variance relation as:

$$\omega' = \omega + \delta^T \Sigma \delta$$

Note that the exact quantification of these quantities is dependent upon the correlation structure between covariates. However, as Σ is positive semi-definite:

$$\omega' \geq \omega$$

This means that the estimated variance of a biased model is larger than the variance of the data.

Appendix B Additional metrics

In addition to the coverage and estimates presented in the main text, we present the additional metrics of the average credible interval width ($\bar{CI}(\theta)$), the standard deviation of the estimates $SD(\theta)$, and average posterior standard deviations $\bar{\epsilon}(\theta)$ for the same parameters of interest across the different replications.

B.1 Practice 1: Ignoring heteroscedasticity

These tables echo the same conclusions as the main text, with the correctly specified model presenting smaller credible interval widths, deviations of the estimate, and average standard errors by an order of magnitude. Increasing numbers of individuals or points also result in improvements across all metrics.

	Models	$SD(\beta^y[Age])$	$\bar{CI}(\beta^y[Age])$	$\bar{\epsilon}(\beta^y[Age])$
100	Correct	0.017	0.063	0.016
300	Correct	0.009	0.035	0.009
500	Correct	0.007	0.026	0.007
1000	Correct	0.005	0.018	0.005

Table B1 Measures of errors of age effect on location for increasing number of individuals.

	Models	$SD(\beta^\omega[Age])$	$\bar{CI}(\beta^\omega[Age])$	$\bar{\epsilon}(\beta^\omega[Age])$
100	Correct	0.024	0.092	0.023
300	Correct	0.013	0.053	0.013
500	Correct	0.010	0.041	0.010
1000	Correct	0.008	0.029	0.007

Table B2 Measures of errors of age effect on scale for increasing number of individuals.

	Models	$SD(\beta^y[Age])$	$\bar{CI}(\beta^y[Age])$	$\bar{\epsilon}(\beta^y[Age])$
5	Correct	0.021	0.082	0.021
10	Correct	0.013	0.055	0.014
20	Correct	0.009	0.037	0.009

Table B3 Measures of errors of age effect on location for increasing number of points.

	Models	$SD(\beta^\omega[Age])$	$\bar{CI}(\beta^\omega[Age])$	$\bar{\epsilon}(\beta^\omega[Age])$
5	Correct	0.039	0.140	0.036
10	Correct	0.022	0.084	0.021
20	Correct	0.014	0.055	0.014

Table B4 Measures of errors of age effect on scale for increasing number of points.

B.2 Practice 2: Misspecifying location and scale in MELSM

Similarly, this section illustrates how different misspecifications affect the different fixed effects with regard to these metrics. Interestingly, the model that considers all covariates and the correctly specified one are indistinguishable from these metrics.

Models	$SD(\beta^y[Age])$	$\bar{CI}(\beta^y[Age])$	$\bar{\epsilon}(\beta^y[Age])$
Correct	0.011	0.043	0.011
All	0.011	0.043	0.011
Mis. ω	0.018	0.056	0.014
No u^ω	0.017	0.065	0.017
No u^ω + Mis. ω	0.044	0.109	0.028

Table B5 Measures of errors of age effect on location.

Models	$SD(\beta^\omega[Age])$	$\bar{CI}(\beta^\omega[Age])$	$\bar{\epsilon}(\beta^\omega[Age])$
Correct	0.016	0.065	0.017
All	0.016	0.065	0.017
Mis. y	0.017	0.068	0.017
No u^ω	0.032	0.055	0.014

Table B6 Measures of errors of age effect on scale.

Models	$SD(\sigma_y)$	$\bar{CI}(\sigma_y)$	$\bar{\epsilon}(\sigma_y)$
Correct	0.054	0.217	0.055
All	0.054	0.218	0.056
Mis. y	0.054	0.218	0.056
Mis. ω	0.058	0.234	0.060
No u^ω	0.055	0.219	0.056
No u^ω + Mis. ω	0.071	0.266	0.068

Table B7 Measures of errors of standard deviation of random intercepts for location.

Models	$SD(\sigma_\omega)$	$\bar{C}I(\sigma_\omega)$	$\bar{\epsilon}(\sigma_\omega)$
Correct	0.031	0.123	0.031
All	0.031	0.124	0.032
Mis. y	0.029	0.109	0.028
Mis. ω	0.041	0.185	0.047

Table B8 Measures of errors of standard deviation of random intercepts for scale.

B.3 Practice 3: Ignoring non-linear time trend in location model

The following tables illustrate these metrics when the correctly specified model depends on the sine of age. Interestingly, these metrics do not capture the bias in the estimated fixed effect, even if the improperly specified model shows narrower parameter estimates. This aligns with the intuition that more complex models may lead to larger standard deviations for the different parameters of interest.

Models	$SD(\beta^y[Age])$	$\bar{CI}(\beta^y[Age])$	$\bar{\epsilon}(\beta^y[Age])$
Correct	0.015	0.058	0.015
Non sinus	0.014	0.047	0.012

Table B9 Measures of errors of age effect on location.

Models	$SD(\beta^\omega[Age])$	$\bar{CI}(\beta^\omega[Age])$	$\bar{\epsilon}(\beta^\omega[Age])$
Correct	0.016	0.065	0.017
Non sinus	0.016	0.065	0.017

Table B10 Measures of errors of age effect on scale.

B.4 Practice 4: Misspecifying the random effect structure

In this setting, the metrics again do not capture the estimate bias, as the correctly specified model presents worse performance on the random effect estimates despite presenting less bias.

The tables show the effect of removing the random slope for age in each of the two submodels. We observe that, once we remove the random slope coefficient from the location submodel, the corresponding fixed effect is affected by low coverage and the estimates of the metrics in the tables are different from those in the correct model. A similar behaviour is observed for the random slope in the scale submodel.

The metrics for the standard deviation of the random intercept in the location and scale submodels remains essentially unaltered after removing the random slopes.

Models	$SD(\beta^y[Age])$	$\bar{CI}(\beta^y[Age])$	$\bar{\epsilon}(\beta^y[Age])$
Correct	0.031	0.120	0.031
No u^{ω}_{age}	0.032	0.121	0.031
No slopes	0.046	0.066	0.017

Table B11 Measures of errors of age effect on location.

Models	$SD(\beta^{\omega}[Age])$	$\bar{CI}(\beta^{\omega}[Age])$	$\bar{\epsilon}(\beta^{\omega}[Age])$
Correct	0.029	0.114	0.029
No u^{ω}_{age}	0.033	0.068	0.017
No slopes	0.032	0.068	0.017

Table B12 Measures of errors of age effect on scale.

Models	$SD(\sigma_y)$	$\bar{CI}(\sigma_y)$	$\bar{\epsilon}(\sigma_y)$
Correct	0.060	0.236	0.060
No u^{ω}_{age}	0.060	0.237	0.061
No slopes	0.053	0.223	0.057

Table B13 Measures of errors of standard deviation of random intercepts for location.

Models	$SD(\sigma_\omega)$	$\bar{CI}(\sigma_\omega)$	$\bar{\epsilon}(\sigma_\omega)$
Correct	0.034	0.132	0.034
No u^{ω}_{age}	0.034	0.129	0.033
No slopes	0.032	0.125	0.032

Table B14 Measures of errors of standard deviation of random intercepts for scale.

B.5 Practice 5: Misspecifying random effect distributions

In this final experiment, the proposed metrics reveal limited differences between the two distribution specifications of the random effects (except for $SD(\sigma^y)$, which is dependent on the different distribution parameterisation). This is consistent with the findings on the limited impact of distribution misspecification.

Models	$SD(\sigma_y)$	$\bar{CI}(\sigma_y)$	$\bar{\epsilon}(\sigma_y)$
Correct	0.147	0.345	0.093
Gaussian	0.190	0.329	0.086

Table B15 Measures of errors of standard deviation of random intercepts for location.

Models	$SD(\sigma_\omega)$	$\bar{CI}(\sigma_\omega)$	$\bar{\epsilon}(\sigma_\omega)$
Correct	0.068	0.190	0.051
Gaussian	0.142	0.182	0.047

Table B16 Measures of errors of standard deviation of random intercepts for scale.
